# Stability of improvements: follow-up data on focused parent–infant psychotherapy (fPIP) for treating regulatory disorders in infancy

**DOI:** 10.1007/s00787-022-02057-9

**Published:** 2022-08-25

**Authors:** Anna Katharina Georg, Markus Moessner, Svenja Taubner

**Affiliations:** 1 Institute for Psychosocial Prevention, University Hospital Heidelberg, Bergheimer Straße 54, 69115 Heidelberg, Germany; 2 Center for Psychotherapy Research, University Hospital Heidelberg, Voßstr. 2, 69115 Heidelberg, Germany

**Keywords:** Early regulatory disorders, Early intervention, Parent–infant psychotherapy, Psychodynamic therapy, Stability of change

## Introduction

Regulatory problems in infancy including persistent excessive crying, sleeping, or feeding difficulties put a child at risk for emotional and behavioral difficulties in childhood and beyond [1]. Research demonstrates that psychodynamic and attachment-based parent–infant psychotherapy (PIP) is effective for psychologically burdened mothers and mother–infant dyads in which the infant has early regulatory disorders (ERD) [2, 3]. Six-month follow-up data showed that 10–18 sessions of PIP maintained or improved outcomes, such as infant symptoms, maternal parenting stress, depression and self-efficacy, and mother–infant interaction [4, 5]. These data suggest that PIP may have positive long-term effects on the child and the family and reduce the likelihood of child emotional and behavioral problems that can emerge when ERD remain untreated. However, several questions regarding the success of early interventions for the treatment of ERD remain open [6] and more research is needed on how psychodynamic and attachment-based interventions affect outcomes, such as parental mental health and parental functions [3].

A type of PIP, focused parent–infant psychotherapy (fPIP) is a brief (four sessions) psychodynamic-based intervention for the treatment of parents with infants at age 4 to 15 months diagnosed with ERD [7, 8]. In an RCT of fPIP versus TAU (treatment as usual; standard pediatric care), fPIP was superior in reducing infants’ overall regulatory symptoms, night-waking disorders, and mothers’ psychological distress and depression after a treatment period of 12 weeks [2]. No intervention effects favoring fPIP were found for emotional availability or parenting stress. Effects on statistical trend level (*p* < 0.10) suggested that fPIP led to increased maternal self-efficacy and parental reflective functioning. While the effects on symptoms in infants and mothers were encouraging, these effects were observed shortly after treatment termination. It is possible that given the brief nature of the treatment, long-term effects on child and parental health outcomes as well as parenting functions cannot be expected.

With the current study, we aimed to investigate whether the improvements made in the fPIP group persisted a year after treatment and if children at follow-up displayed normative child functioning. Our hypothesis was that improvements on mothers’ psychological distress, depression, level of parenting stress, self-efficacy and reflective functioning [2] would remain stable at the follow-up. In addition, we expected that children in the fPIP group would not show elevated emotional and behavioral problems or sleep problems compared to norm or comparison samples. Sleep problems were considered as an outcome as sleep onset (64%) and night-waking disorders (85%) were the most frequent diagnosis in the sample participating in the RCT.

## Method

The approval for this research was obtained from Medical Faculty of Heidelberg University (No. S-541/2013 approved November 4, 2013).

### Procedure

Families who had received fPIP within the RCT [2] were contacted by a research assistant for the follow-up assessment (T3) one year after the post-test (T2) had taken place. If they agreed to participate, they were sent a set of questionnaires. Families from the TAU-group were not considered, because an unexpected high number of families (44.6%) were referred to a PIP-intervention following completion of T2 due to ongoing high distress in the family and thus were not asked to participate in T3.

### Instruments

Details about the instruments utilized to assess intervention outcomes on psychological distress (Symptom-Checklist-90R-S, SCL-GSI) and depression (SCL-DE) parenting stress (Parenting Stress Index, PSI), maternal self-efficacy (Maternal-Self-Efficacy-Scale, MSES), and parental reflective functioning (Parental Reflective Functioning Questionnaire, PRFQ) can be obtained from [2].

To estimate normative child functioning at T3, age-appropriate instruments on child emotional and behavioral problems and particularly sleeping problems were utilized.

The Child Behavior Checklist (CBCL) assesses parental reports of a child’s emotional and behavior problems [9]. Reference *T*-scores for internalizing and externalizing problems and the total score are provided by a community sample of healthy U.S. children aged 18–71 months [9]. In the German CBCL ½–5, the total (CBCL-total), internalizing (CBCL-IN), and externalizing (CBCL-EX) scores yielded good internal consistencies (Cronbach’s Alpha = 0.85–0.95) and the factorial validity was confirmed [10].

The Brief Infant Sleep Questionnaire (BISQ) [11] assesses parent-reported sleep patterns, sleep ecology, and parental perceptions of sleep and was translated into German (translation/back-translation) for this study. The clinical screener consists of the indicators: > 3 wakings per night, nocturnal wakefulness > 1 h, and total sleep duration < 9 h [11, 12]. The screener correctly classified 80% of children in control or clinical samples [11] and predicted objective sleep measures [12].

### Statistical analysis

To test the hypothesis that the effects of fPIP persist at T3, we conducted several multi-level models (MLM) with time points (level 1) nested within participants (level 2). Every model consisted of categorical time (T1, T2, and T3) at level 1 as a predictor. We utilized *Q*–*Q* plots and sphericity plots to examine if the assumptions of MLM were met and no significant deviations were found.

The Reliable Change Index [13] was used to determine the proportion of improvement and deterioration deemed significantly reliable from T1 to T3. Only those variables with available test–retest reliability indicators were chosen for the analysis which were SCL-GSI, SCL-DE, and PSI scores. Reliable change on child symptomatology could not be analyzed due to the use of different age-appropriate instruments from T1 to T3.

Missing data (0.58%) were imputed using R version 3.5.2 [14] and assuming missing values at random by utilizing multiple imputation by chained equations with 40 iterations [15]. One multivariate outlier was found and thus excluded from analysis: Mahalanobis’ distance had a *χ*^2^ critical value of 24.37 (*p* < 0.001). SPSS version 27 [16] was used for the main data analysis.

## Results

### Study participants

The participant flow from enrollment to T2 can be found in [2]. Among the 81 families randomized to fPIP at T1, 75 (93%) participated in T2 and 53 (65%) in T3. Table 1 describes sample characteristics of the T3 sample as well as the total fPIP group at baseline (T1). By the time of T3, children’s ages ranged between 19 and 39 months (*M* = 26.14; SD = 3.89).

**Table 1:** Demographic and clinical characteristics of the T3 sample (*n* = 52) of the fPIP group and the entire fPIP sample of the RCT (*n* = 81) at T1 and drop-out analysis

Characteristic	fPIP (*n* = 81)	Sample T3 (*n* = 52)	Drop-out analysis (sample T3 vs. drop-out sample T3)
Infant			
Female child, *n* (%)	38 (46.91)	27 (51.90)	*χ*^2^(1) = 1.166, *p* = .280
Age, *M* (*SD*)	8.84 (3.20)	9.13 (3.28)	*t*(78) = 1.089, *p* = .280
First born child, *n* (%)	49 (60.49)	29 (55.80)	*χ*^2^(1) = 1.628, *p* = .202
Diagnosis			
Persistent excessive crying, *n* (%)	8 (9.88)	2 (3.80)	*χ*^2^(1) = 6.252, *p* = **.012**
Sleep onset disorder, *n* (%)	53 (65.43)	32 (61.50)	*χ*^2^(1) = 1.475, *p* = .225
Night-waking disorder, *n* (%)	71 (87.65)	48 (92.30)	*χ*^2^(1) = 3.140, *p* = .076
Regulation disorder of sensory processing, *n* (%)	42 (51.85)	26 (50.00)	*χ*^2^(1) = 372, *p* = .542
Feeding disorder, *n* (%)	11 (13.58)	5 (9.60)	*χ*^2^(1) = 2.142, *p* = .143
Sum of regulatory disorders, median (min; max)	2 (1; 5)	2 (1; 4)	*U* = 584.00, *p* = .123
PIR-GAS, *M* (*SD*)	7.37 (1.02)	7.56 (.98)	*t*(78) = 2.397, *p* = **.019**
Mother			
Age, *M* (*SD*)	33.10 (4.40)	33.55 (4.44)	*t*(78) = 1.398, *p* = .166
Mother has High School or higher education, *n* (%)	59 (72.84)	40 (76.90)	χ^2^(1) = 1.458, *p* = .227
Mother married, *n* (%)	64 (79.01)	41 (78.80)	χ^2^(1) = .001, *p* = .977
Mother of German origin, *n* (%)	73 (90.12)	48 (92.3)	χ^2^(1) = 257, *p* = .612

Diagnosis according to DC: 0–3R or AWMF-guidelines (cf. Georg et al. [2])

*fPIP* focused parent–infant psychotherapy, *PIR-GAS* parent–infant relationship global assessment scale

Compared to the drop-out sample (*n* = 28), the T3 sample (*n* = 53) presented with higher Parent-infant Relationship Global Assessment (PIR-GAS) scores (T3 sample: *M* = 7.56, SD = 0.98 vs. drop-out sample: *M* = 7.00, SD = 1.02) and fewer infants diagnosed with persistent excessive crying disorder at T1 (T3 sample: *n* = 2 vs. drop-out sample: *n* = 6) (Table 1). On average, the T3 and the drop-out sample were classified in the same clinical range of the PIR-GAS (7.1–8.0, perturbed) and values in the T3 and drop-out sample ranged between minimum 5.0 (disturbed) and maximum 9.0 (adapted). 

### Stability of change in outcome measures

Table 2 displays the HML results. Descriptive statistics on each time point are available in the online supplement. Data show significant changes between T1 and T2 except for the PRFQ-IC scale. Scores remained stable from T2 to T3 on all outcome measures (*p*’s > 0.132) meaning that all significant changes observed immediately after treatment persisted at follow-up.

**Table 2:** Multilevel models on outcome variables in the T3 sample (*n* = 52) of the fPIP group

Predictors	SCL-GSI		SCL-DE		PSI		MSES		PRFQ-PM^a^		PRFQ-IC^a^		PRFQ-CMS^a^	
	Estimate [95% CI]	*p*	Estimate [95% CI]	*p*	Estimate [95% CI]	*p*	Estimate [95% CI]	*p*	Estimate [95% CI]	*p*	Estimate [95% CI]	*p*	Estimate [95% CI]	*p*
(Intercept)	0.31 [0.22; 0.39]	** < .001**	6.17 [4.40; 7.94]	** < .001**	121.65 [113.30; 130.00]	** < .001**	34.00 [33.04; 34.96]	** < .001**	1.37 [1.19; 1.56]	** < .001**	4.96 [4.69; 5.24]	** < .001**	4.48 [4.17; 4.80]	** < .001**
T2 vs. T1	0.22 [0.15; 0.29]	** < .001**	4.67 [2.87; 6.48]	** < .001**	11.21 [4.54; 17.88]	**.001**	− 1.52 [− 2.43; − 0.61]	**.001**	0.23 [0.05; 0.41]	**.015**	0.12 [− 0.18; 0.43]	.424	− 0.39 [− 0.73; − 0.06]	.**022**
T2 vs. T3	0.01 [− 0.06; 0.08]	.708	0.54 [− 1.27; 2.32]	.555	1.88 [− 4.79; 8.56]	.577	0.23 [− 0.68; 1.14]	.617	0.14 [− 0.04; 0.32]	.132	0.03 [− 0.28; 0.33]	.856	0.03 [− 0.31; 0.36]	.869

The bold values (*p*-values) point to a significant effect (estimate)

The reference category for time is T2 to best showcase the change in outcome variables

*fPIP* focused parent–infant psychotherapy, *MSES* Maternal Self-Efficacy Scale, *PRF-CMS* Certainty of Mental States scale of the Parental Reflective Functioning Questionnaire, *PRF-IC* Interest and Curiosity scale of the Parental Reflective Functioning Questionnaire, *PRF-PM* Prementalizing Scale of the Parental Reflective Functioning Questionnaire, *PSI* Parenting Stress Index, *ERD* Early Regulatory Disorders, *SCL-DE* Depression scale of the Symptom-Check-List-90R-S, *SCL-GSI* General Severity Index of the Symptom-Check-List-90R-S

^a^Sample size with PRFQ scores was smaller (T1: *n* = 48; T2: *n* = 48, T3: *n* = 30)

### Reliable change

At T3 compared to T1, mothers reliable improved in the PSI (25%), the SCL-GSI (26.92%), and the SCL-DE (21.15%). Reliable deterioration was observed for three mothers (5.77%) in the PSI and for one mother (1.92%) in the SCL-GSI and the SCL-DE.

### Child functioning

Child CBCL-total scores at T3 (*M* = 22.19, SD = 16.04) ranged from *T* = 28 to 72 (*M*_*T*_ = 43.87, SD_*T*_ = 8.98). One child scored above the clinical cut-off (*T* ≥ 64). CBCL-IN scores (*M* = 4.63, SD = 5.10) ranged from *T* = 29 to 68 (*M*_*T*_ = 41.87, SD_*T*_ = 10.30) with two children scoring in the clinical range. CBCL-EX scores (*M* = 9.25, SD = 6.90) ranged from *T* = 28 to 73 (*M*_*T*_ = 45.35, SD_*T*_ = 8.99) and three children scored above the cut-off.

According to the BISQ clinical screener, seven children (13.50%) were screened as having clinically significant sleeping problems.

## Discussion

This study investigated if improvements made by children and their mothers observed at the end of a brief intervention for ERD persisted at follow-up. Overall, our results point to a continued stable family situation a year post-treatment. The short-term intervention effects in favor of fPIP on maternal psychological distress, depression, maternal self-efficacy, and prementalizing [2], were maintained at follow-up. In addition, significant short-term improvements in the fPIP group on parenting stress and parental mentalizing persisted at T3. Although deterioration effects were found in some cases, a number of relatively large positive improvement rates were found with regards to maternal parenting stress, psychological distress and depression.

Children overall demonstrated good emotional and behavioral and sleeping outcomes at T3. Compared to a German community sample [10], overall problems and internalizing problems were less frequent (CBCL-total: *M* = 29.17, SD = 19.51; CBCL-IN: *M* = 8.48, SD = 6.73), while externalizing problems were similar (CBCL-EX: *M* = 10.84, SD = 7.61). Only seven (13.46%) children were screened positively for sleeping problems, which is significantly less than the prevalence rates in community samples (e.g., 36% in 24-month-old children [12] and 34.15% in 24–30-month-old children [11]). These results suggest that fPIP may help to reduce the risk of developing emotional and behavioral problems and sleeping problems in 19–39-month-old children who were previously diagnosed with ERD.

Overall our results add to previous literature on follow-up data of PIP in high-risk families [4, 5] and point to the persistency of effects on child and mother outcomes a year post treatment from a brief intervention. Given the deterioration effects in maternal parenting and psychological distress observed in some cases (7.69%), it is possible that a brief treatment focusing on the child and the mother–child relationship may not be sufficient to achieve a long-term stabilization of these parameters in some families. Future research in larger samples may focus on the moderators that explain why some cases do not benefit from the intervention. 

The small effects of improvements on maternal self-efficacy and parental reflective functioning may further bring about a long-lasting stabilization of parenting resources that have been shown to contribute to a better parent–child relationship [17]. Mothers who participated with their child in fPIP were likely better able to respond to their children’s needs beyond the problematic situations related to ERD and may have felt more secure in parenting their child.

This study is limited by the lack of a control group for T3. Thus, we cannot conclude that the stability of outcomes from T2 to T3 was specific to the intervention. The families who dropped out at T3 (34.57%) presented to treatment with more infants diagnosed with persistent excessive crying disorders and with lower quality parent–infant relationships. However, the difference in the PIR-GAS was not clinically meaningful and both samples on average were rated as having perturbed relationship qualities. Thus, our results may not generalize to all at-risk families, particularly not to those with children with persistent excessive crying disorders.

This study underlines the potential of brief psychodynamic PIP to reduce the childhood emotional and behavioral problems that can follow from untreated ERD. The results apply to a sample of comorbid ERD with a predominance of night-waking disorders and minor to major mother–infant relationship problems and need further replication in more high-risk, diverse samples.

### Supplementary Information

Below is the link to the electronic supplementary material.

Supplementary file1 (DOCX 16 KB)

## Declarations

### Clinical trial registration information

The efficacy of a brief parent-infant psychotherapy for the treatment of early regulatory disorders: A randomized controlled trial; https://www.drks.de/drks_web/; DRKS00005739. Date of registration: 19.03.2014. Start of data collection: 03.02.2014.

### Data transparency

The data that support the findings of this study are available on request from the corresponding author. Research data cannot be made publicly available due to legal restrictions.
