# Supplementary material for: Stability of improvements: follow-up data on focused parent–infant psychotherapy (fPIP) for treating regulatory disorders in infancy
Source: Eur Child Adolesc Psychiatry. 2022 Aug 25;32(11):2379–83. doi: 10.1007/s00787-022-02057-9 (PMC10576718; doi:10.1007/s00787-022-02057-9)
Supplement: Supplementary file 1 — Supplementary file1 (DOCX 16 KB) [file 787_2022_2057_MOESM1_ESM.docx]

**Supplement**

*Descriptive statistics on each measurement point of the T3 sample (n = 52) in the fPIP group*

| Outcome variable | **T1** | **T2** | **T3** |
| --- | --- | --- | --- |
|  | ***M* (*SD*)** | ***M* (*SD*)** | ***M* (*SD*)** |
| SCL-GSI | .529 (.33) | .307 (.26) | .320 (.32) |
| SCL-DE | 10.85 (7.04) | 6.17 (5.97) | 6.71 (6.25) |
| PSI | 132.87 (28.61) | 121.65 (30.33) | 123.54 (31.74) |
| MSES | 32.48 (3.86) | 34.00 (3.10) | 34.23 (3.42) |
| PRF-PM^a^ | 1.60 (.59) | 1.37 (.38) | 1.51 (.52) |
| PRF-IC^a^ | 5.08 (.79) | 4.96 (.78) | 4.99 (.70) |
| PRF-CMS^a^ | 4.09 (.87) | 4.48 (.90) | 4.51 (.84) |

*Note.* fPIP = focused parent-infant psychotherapy; MSES = Maternal Self-Efficacy Scale; PRF-CMS = Certainty of Mental States scale of the Parental Reflective Functioning Questionnaire; PRF-IC = Interest and Curiosity scale of the Parental Reflective Functioning Questionnaire; PRF-PM = Prementalizing Scale of the Parental Reflective Functioning Questionnaire; PSI = Parenting Stress Index; SCL-DE = Depression scale of the Symptom-Check-List-90R-S; SCL-GSI = General Severity Index of the Symptom-Check-List-90R-S.

^a^ = Sample size with PRFQ scores was smaller (T1: *n* = 48; T2: *n* = 48, T3: *n* = 30).
